# Still on the Brazilian Response to the Microcephaly Epidemic: A Meta-analysis of 1,548 Pregnant Women from 13 Cohorts to Evaluate the Risk of Adverse Outcomes

**DOI:** 10.1055/s-0043-1769107

**Published:** 2023-05-24

**Authors:** Ricardo Arraes de Alencar Ximenes, Demócrito de Barros Miranda-Filho, Flor Ernestina Martinez-Espinoza, Patrícia Brasil

**Affiliations:** 1 Universidade Federal de Pernambuco, Recife, PE, Brazil; 2 Universidade de Pernambuco, Recife, PE, Brazil; 3 Universidade do Estado do Amazonas, Manaus, AM, Brazil; 4 Fundação Oswaldo Cruz, Manguinhos, RJ, Brazil

In 2015, the scientific community was surprised by an epidemic of microcephaly initially identified in some states in northeastern Brazil. The first observations of an unusual increase in the number of cases of microcephaly were made by physicians in their clinical practice. After confirming the occurrence of this new phenomenon, came the challenges in determining its etiology, characterizing the spectrum of clinical manifestations and estimating the risk of its occurrence. These stages were successively fulfilled through ecological studies, case reports and series, and epidemiological studies. 1 Clinicians were the first to raise the hypothesis that Zika virus infection during pregnancy was responsible for the adverse effects observed in children. 2 Subsequently, the virus was detected and sequenced in the amniotic fluid of two pregnant women whose fetuses had microcephaly 3 and specific IgM for Zika was detected in the cerebrospinal fluid of children with microcephaly. 4 A case-control study showed the association between the Zika virus and microcephaly and at the same time, ruled out the role of other factors that could be responsible for its occurrence. 5 The follow-up of cohorts of pregnant women allowed estimating the risk for microcephaly, abnormalities of the Central Nervous System (CNS) diagnosed by imaging, ophthalmologic and audiologic alterations and other birth defects in children born to Zika virus-infected mothers during pregnancy. 6 7 8 9 10 11 12 13 Although cohort studies have shown similar risks of microcephaly, estimates of the risk of other manifestations were diverse, indicating the need to use other analysis strategies with more robust estimates, such as meta-analysis.

In Brazil, cohort studies were developed by different groups of researchers. However, since the beginning of the microcephaly epidemic, Brazilian scientists were concerned about standardizing research protocols and collection instruments as far as possible to enable a joint data analysis in a later step. Several meetings were held to this end, initially involving Brazilian researchers and later researchers from different countries, with support of the Pan American Health Organization and the World Health Organization. In Brazil, the Zika Brazilian Cohorts (ZBC) Consortium 14 was formed. By performing a joint analysis of data from Brazilian studies, it overcomes the limitations of isolated studies, notably the small sample size and consequent inaccuracy of estimates and lower representativeness. Among the contributions of the ZBC Consortium is the recently published article: “Risk of adverse outcomes in offspring with RT-PCR confirmed prenatal Zika virus exposure: an individual participant data meta-analysis of 13 cohorts in the Zika Brazilian Cohorts Consortium.” 15 Next, we will highlight some of its points.

Several factors reinforce the relevance of the results presented in this article. It is a meta-analysis of individual data that aggregates and analyzes data from different studies after a process of harmonization of results. Harmonization was performed through several meetings of researchers and enabled the formation of a single database and the analysis of information from all participants, differing from traditional meta-analyzes in which only aggregated data are reanalyzed. The Consortium included almost all cohorts of pregnant women developed in Brazil, totaling 13 studies performed in four Brazilian regions where the Zika virus epidemic occurred, namely the North, Northeast, Central West and Southeast. It is the study with the largest number of participants published so far, totaling 1,548 pregnant women and their respective gestational outcomes. All women had Zika virus infection during pregnancy confirmed through RT-PCR, the gold standard for diagnosing Zika virus infection. 16 Because of interpretation limitations, serological tests were not used to define exposure.

The results of this ZBC-Consortium meta-analysis provide more robust and accurate estimates of the risk of adverse events in children born to pregnant women who were infected with Zika virus during pregnancy. This study answers an important question for physicians and health professionals by informing the probability of occurrence of manifestations potentially associated with congenital Zika.

According to the study findings, although microcephaly is the most severe manifestation, it is not the most frequent, being observed in 1.5% of children at birth, and severe microcephaly is less frequent than mild/moderate microcephaly. Furthermore, even though some children are born with a normal head circumference for their age and sex, they may develop postnatal microcephaly, which implies the need to monitor these children and repeat head circumference measurements. It was also demonstrated that the risk of children being born small for gestational age was greater than the risk reported for the general population. Unlike what had been suggested by some authors, the risk of microcephaly did not vary in different regions of the country or with different socioeconomic conditions.

The risk of occurrence of structural changes in the CNS in children born to mothers who became infected during pregnancy was around 8%, and was observed even in children without microcephaly. The most frequent were calcifications, ventriculomegaly and diffuse cortical atrophy, in addition to other manifestations identified. Ultrasound imaging of the CNS after birth is a valuable tool for diagnosing structural alterations.

The risk of presenting at least one neurological alteration was around 20%, highlighting the occurrence of changes in tonus/trophism and convulsive crises. The risk of these alterations (20%) was greater than that of microcephaly and structural abnormalities in the CNS, showing the complementarity of this information and the need to integrate them for an adequate and long-term evaluation of these children.

The risks of audiological and ophthalmological adverse effects, especially changes in the optic nerve, were less than 5%.

Approximately one-third of infants born to mothers exposed to the Zika virus during pregnancy had at least one change, and less than 1% had concomitant changes.

The risks estimated in the ZBC-Consortium meta-analysis are relevant for planning care for pregnant women who become infected with the Zika virus during pregnancy and the care for children born to these mothers. Note that the possibility of a new Zika virus epidemic cannot be ruled out as the number of susceptible individuals increases. The study highlights the need for at least one comprehensive assessment of children by different groups of specialists during their follow-up for the early detection of abnormalities and definition of the necessary interventions. The study also indicates the need for long-term monitoring of children to identify the risk of late manifestations.
